# Everolimus (RAD001) sensitizes prostate cancer cells to docetaxel by down-regulation of HIF-1α and sphingosine kinase 1

**DOI:** 10.18632/oncotarget.13115

**Published:** 2016-11-04

**Authors:** Heba Alshaker, Qi Wang, Yoshiaki Kawano, Tawfiq Arafat, Torsten Böhler, Mathias Winkler, Colin Cooper, Dmitri Pchejetski

**Affiliations:** ^1^ School of Medicine, University of East Anglia, Norwich, UK; ^2^ Department of Pharmacology and Biomedical Sciences, Faculty of Pharmacy and Medical Sciences, University of Petra, Amman, Jordan; ^3^ Department of Urology, University of Kumamoto, Kumamoto, Japan; ^4^ Department of Pharmaceutical Medicinal Chemistry and Pharmacognosy, Faculty of Pharmacy and Medical Sciences, University of Petra, Amman, Jordan; ^5^ Department of Surgery and Cancer, Imperial College London, London, UK

**Keywords:** prostate cancer, everolimus (RAD001), mTOR, docetaxel, chemosensitization

## Abstract

Resistance to docetaxel is a key problem in current prostate cancer management. Sphingosine kinase 1 (SK1) and phosphoinositide 3-kinase (PI3K)/Akt/mammalian target of rapamycin (mTOR) pathways have been implicated in prostate cancer chemoresistance. Here we investigated whether their combined targeting may re-sensitize prostate cancer cells to docetaxel.

In hormone-insensitive PC-3 and DU145 prostate cancer cells the mTOR inhibitor everolimus (RAD001) alone did not lead to significant cell death, however, it strongly sensitized cells to low levels (5 nM) of docetaxel. We show that mTOR inhibition has led to a decrease in hypoxia-inducible factor-1α (HIF-1α) protein levels and SK1 mRNA. HIF-1α accumulation induced by CoCl_2_ has led to a partial chemoresistance to RAD001/docetaxel combination. SK1 overexpression has completely protected prostate cancer cells from RAD001/docetaxel effects. Using gene knockdown and CoCl_2_ treatment we showed that SK1 mRNA expression is downstream of HIF-1α. In a human xenograft model in nude mice single RAD001 and docetaxel therapies induced 23% and 15% reduction in prostate tumor volume, respectively, while their combination led to a 58% reduction. RAD001 alone or in combination with docetaxel has suppressed intratumoral mTOR and SK1 signaling, however as evidenced by tumor size, it required docetaxel for clinical efficacy. Combination therapy was well tolerated and had similar levels of toxicity to docetaxel alone.

Overall, our data demonstrate a new mechanism of docetaxel sensitization in prostate cancer. This provides a mechanistic basis for further clinical application of RAD001/docetaxel combination in prostate cancer therapy.

## INTRODUCTION

Prostate cancer is now the most frequently diagnosed cancer among men in developed countries and the second most common cause of cancer related mortality [1]. Androgen suppression is the principal initial systemic therapy for metastatic prostate cancer [2]. However, inherent or acquired resistance to androgen therapy remains a major clinical obstacle [3] and eventually most patients with advanced disease relapse [4]. Docetaxel chemotherapy offered to these patients only extends survival for a median period of less than 3 months [5]. The data of GETUG-AFU 15 [6] and STAMPEDE [7] clinical trials have revitalized interest in docetaxel. It was found that in men with hormone-sensitive metastatic prostate cancer early docetaxel administration combined with androgen deprivation therapy statistically significantly improved overall survival by 10 months, compared with androgen deprivation therapy alone [7]. Considering that docetaxel effect on the median overall survival in metastatic castration-resistant prostate cancer (mCRPC) is several-fold less than its impact in hormone-sensitive metastatic prostate cancer, it is imperative to identify potential chemotherapy targets that might sensitize mCRPC cells to taxane therapies.

The loss of phosphatase and tensin homologue (PTEN) deleted on chromosome 10 is prevalent in the majority of advanced prostate cancers leading to constitutive activation of the phosphoinositide 3-kinase (PI3K)/Akt/mammalian target of rapamycin (mTOR) pathway [8]. Many inhibitors of the PI3K/Akt/mTOR pathway have shown activity in preclinical cancer models [9, 10]. An mTOR inhibitor CCI-779 (temsirolimus) was previously shown to potentiate chemotherapy effects in prostate cultures [11], however the exact mechanism beyond the mTOR inhibition was not elucidated in that study. Of particular interest is an orally bioavailable mTOR complex 1 (mTORC1) inhibitor, everolimus (RAD001) [12], which is approved for treatment of metastatic renal-cell carcinoma [13]. In prostate cancer RAD001 was shown to induce cancer cell apoptosis and to completely reverse neoplasms in mice expressing human Akt1 in their prostates [14]. However, single-agent mTOR inhibitors (including RAD001) demonstrated low level of clinical activity in men with mCRPC [15–17]. The potential mechanism of this resistance may be explained by a rebound activation of upstream Akt [15]. To overcome resistance, investigators have tired combining mTOR inhibition with tubulin depolymerization by docetaxel and have successfully reduced mCRPC metastasis in mice [18]. The same combination is under investigation in clinical trials [19].

Resistance to docetaxel is a common problem for the treatment of mCRPC. Our previous data showed a critical role of a lipid kinase sphingosine kinase 1 (SK1) in prostate cancer chemoresistance, and introduced the concept of SK1 as a “sensor” to anticancer therapies [20]. We have shown a significant radio- and chemosensitizing potential of SK1 inhibition in mCRPC, particularly when combined with docetaxel chemotherapy [20–22]. In cell and animal prostate cancer models, silencing of SK1 decreases cancer cell migration and invasion and resistance to docetaxel [20–22].

This current study is set with the aim of exploring the effect of the combination of RAD001 with conventional chemotherapeutic drug, docetaxel, on prostate cancer cells *in vitro* and *in vivo.* We have investigated the mTOR-mediated regulation of hypoxia-inducible factor-1α (HIF-1α) and SK1 pathways providing a mechanistic basis for further clinical application of RAD001 in prostate cancer therapy.

## RESULTS

### RAD001 sensitizes PC-3 cells to docetaxel

**Figure 1:**
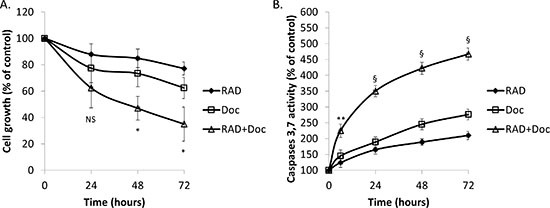
RAD001 sensitizes prostate cancer cells to small doses of docetaxel PC-3 cells were starved overnight then incubated with 0.1% DMSO (Cont), 100 nM RAD001 (RAD), 5 nM docetaxel (Doc) and the combination of these drugs (RAD+Doc) for 72 h. (**A)** Cell proliferation was measured using MTT assay. **(B)** Activity of caspases 3,7 was measured using caspases assay. *Points,* mean of three independent experiments performed in triplicate; *bars*, SEM. (*P < 0.05; **P < 0.01; ^§^*P* < 0.001; NS, not significant, *P* > 0.05).

RAD001 at 100 nM mildly reduced PC-3 and DU145 cell viability in a time-dependent manner (Figure 1A; Supplementary Figure S1A). This effect was significantly increased when it was combined with 5 nM docetaxel. In PC-3 cells, at 72 hour (h) individual RAD001 and docetaxel induced 23% and 38% reduction in cell viability, respectively, while the combination of both drugs induced a 65% reduction in cell viability (Figure 1A).

Similarly to cell viability, both RAD001 and docetaxel have time-dependently induced activation of caspases 3 and 7 in both cell lines (Figure 1B; Supplementary Figure S1B). In PC-3 cells at 6 h, RAD001 and docetaxel individually induced a moderate increase in caspases 3,7 activity of 124% and 145%, respectively, while their combination induced a 225% increase (Figure 1B). The maximum increase of 467% was achieved by the drug combination at 72 h while the individual drugs could only achieve ~200–250% increase (Figure 1B). A similar pattern was shown by DU145 cells (Supplementary Figure S1B). Of note, our previous studies showed that 20 nM docetaxel is required to successfully induce apoptosis in PC-3 cells as a single therapy [20, 22], therefore 5 nM dose represents a significant (4-fold) reduction in effective dose. Overall, our findings suggest that RAD001 is a potent sensitizer to low doses of docetaxel in prostate cancer cell culture models.

### RAD001 down-regulates mTOR-dependent HIF-1α accumulation and decreases SK1 expression

**Figure 2:**
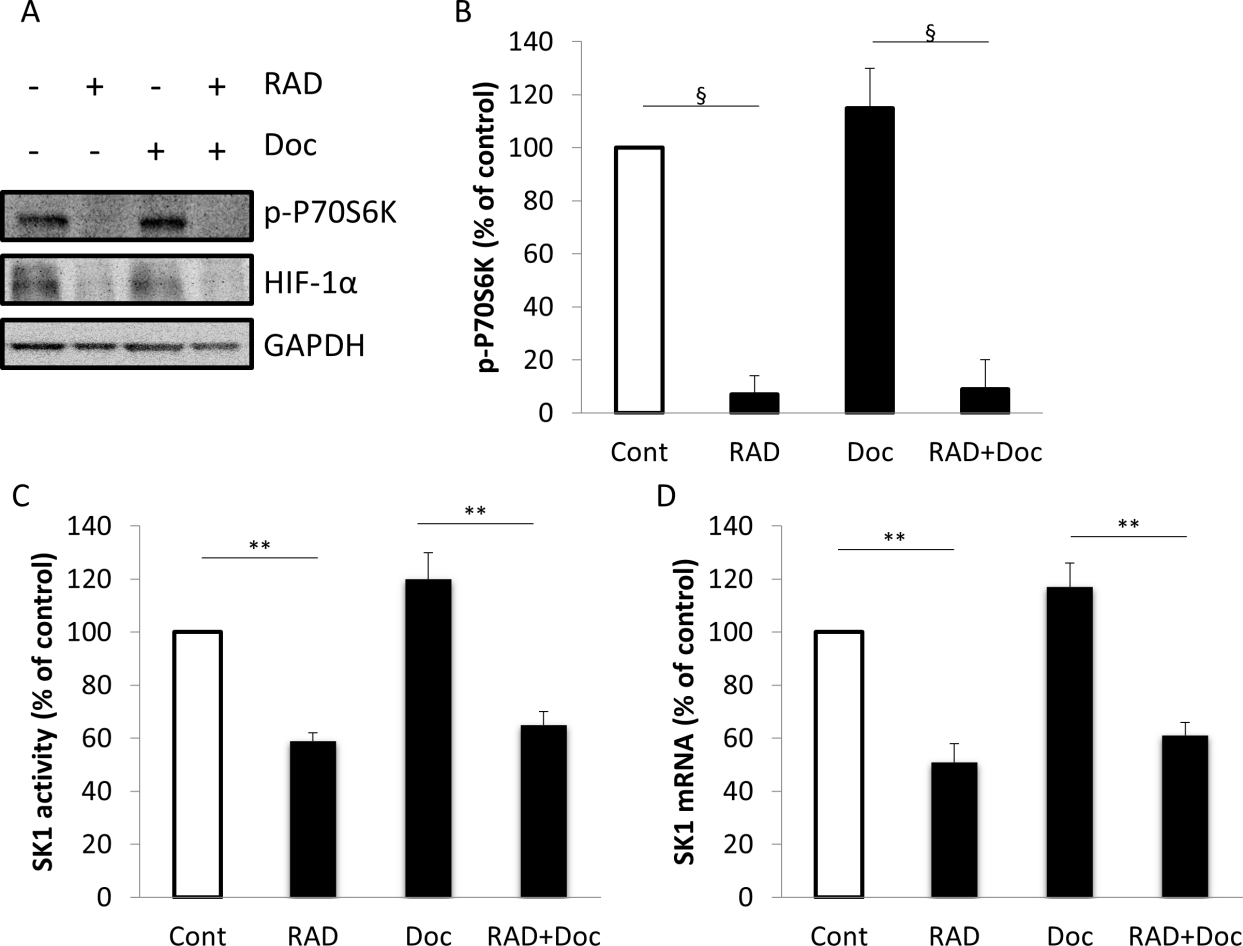
RAD001 decreases P70S6K phosphorylation, HIF-1α protein levels, SK1 expression and activity PC-3 cells were starved overnight then incubated with 0.1% DMSO (Cont), 100 nM RAD001 (RAD), 5 nM docetaxel (Doc) and the combination of these drugs (RAD+Doc) for 24 h. (**A)** Cell extracts were loaded on acrylamide mini gel and probed for phosphorylation of P70S6K, HIF-1α and GAPDH. (**B)** P70S6K phosphorylation was measured using ELISA. (**C)** SK1 activity was measured using radiolabeling. (**D)** SK1 expression was determined by qRT-PCR, normalized against housekeeping genes (GAPDH, YWHAZ and UBC) and analyzed using qBase software. Columns, mean of three independent experiments performed in triplicate; bars, SEM. (*P < 0.05; **P < 0.01; ^§^*P* < 0.001; NS, not significant, *P* > 0.05).

We had previously established that sustained SK1 expression and its enzymatic activity mediate resistance to docetaxel in prostate cancer [22]. It has been suggested that in leukemic cells PI3K/Akt/mTOR pathway may increase SK1 levels [23]. In human prostate cancer cell line PC-3, mTOR signaling pathway is an upstream activator of HIF-1 [24]. SK1 has been reported as a downstream target of HIF-1α in other systems [25, 26], whereas in prostate cancer this relationship was not investigated. To determine whether all these mechanisms exist in prostate cancer cells, we examined the levels of phosphorylated (p)-P70S6 Kinase (P70S6K) and HIF-1α in PC-3 and DU145 cells (Figure 2A; Supplementary Figure S2A) treated for 24 h with 5 nM docetaxel and 100 nM RAD001.

It is well established that mTORC1 directly phosphorylates P70S6K on Thr389 (a residue critical for S6 kinase activity) [27] and this phosphorylation of P70S6K at Thr389 has been widely used as a surrogate for mTORC1 activity [28]. Western blot analysis showed that while docetaxel did not decrease p-P70S6K and HIF-1α protein levels in either PC-3 or DU145 cells, RAD001 has reduced both p-P70S6K and HIF-1α levels both alone and in combination with docetaxel (Figure 2A; Supplementary Figure S2A). We have also used an ELISA assay to analyze P70S6K phosphorylation, which showed similar results (Figure 2B; Supplementary Figure S2B). Suppression of mTOR activity by RAD001 either alone or in combination with docetaxel significantly decreased SK1 mRNA levels and enzymatic activity (Figure 2C, 2D). Comparable findings were obtained in DU145 cells (Supplementary Figure S2C, S2D). Our data indicate that in prostate cancer cells 5 nM docetaxel does not inhibit mTORC1 activity (as assessed by p-P70S6K), HIF-1α protein levels, SK1 activity and expression and that combining docetaxel with RAD001 allows a marked reduction in these signaling pathways (Figure 2; Supplementary Figure S2) and chemosensitization (Figure 1; Supplementary Figure S1).

### Overexpression of SK1 restores prostate cancer cells chemoresistance

**Figure 3:**
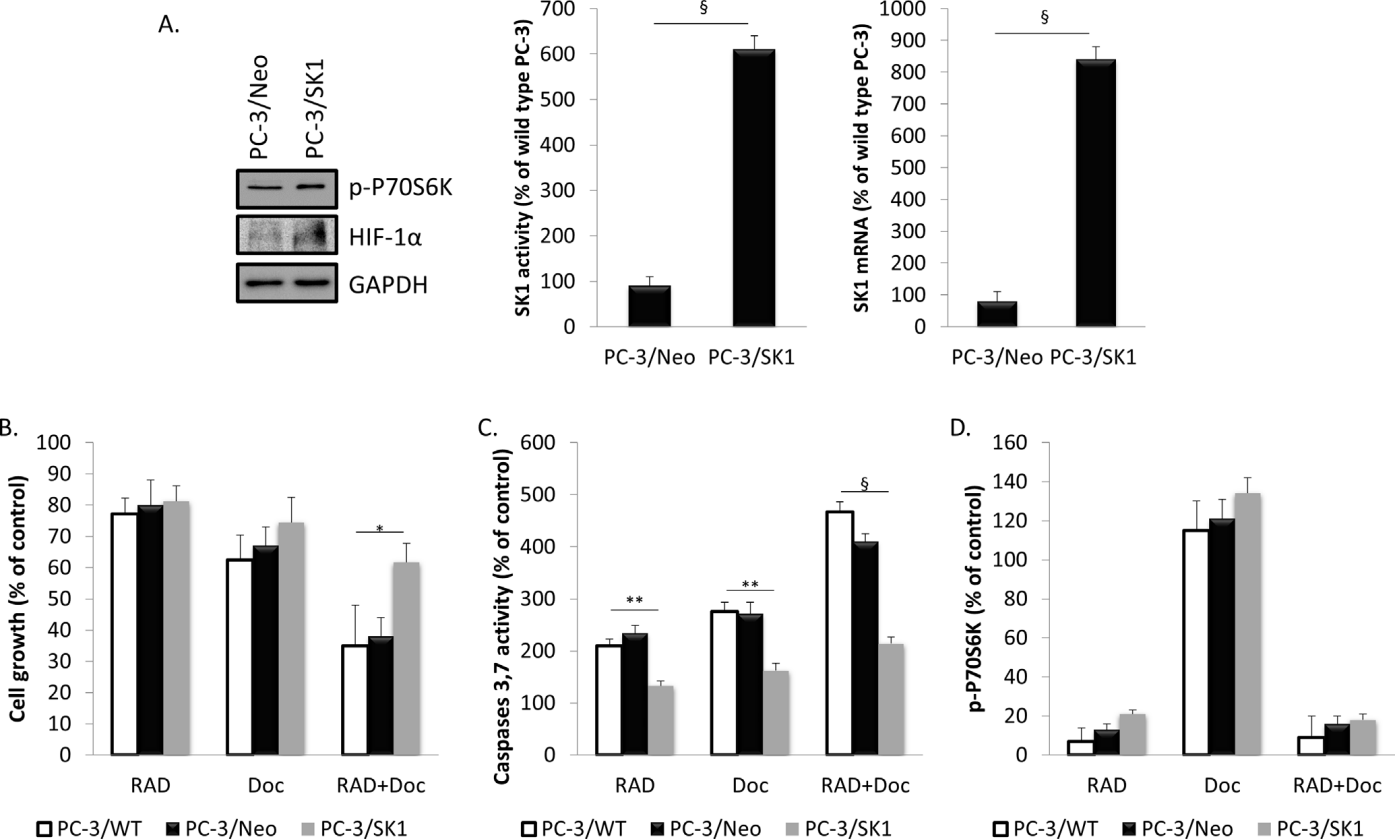
Overexpression of SK1 restores prostate cancer cells chemoresistance without affecting RAD001-induced mTOR inhibition (**A**) Cell extracts for empty vector-transfected PC-3 cells (PC-3/Neo) and PC-3 cells stably transfected with human SK1 (PC-3/SK1) were loaded on acrylamide mini gel and probed for p-P70S6K, HIF-1α and GAPDH; SK1 activity and SK1 expression were measured using radiolabeling and qRT-PCR, respectively. Wild type PC-3 (PC-3/WT), PC-3/Neo and PC-3/SK1 cells were starved overnight then incubated with 0.1% DMSO (Control), 100 nM RAD001 (RAD), 5 nM docetaxel (Doc) and the combination of these drugs (RAD+Doc) for 24–72 h (**B–D**). (B) Cell proliferation was measured using MTT assay at 72 h. (C) Activity of caspases 3,7 was measured using caspases assay at 72 h. (D) P70S6K phosphorylation was measured using ELISA at 24 h. Data (B-D) is expressed as percent relative to relevant untreated controls. Columns, mean of three independent experiments performed in triplicate; bars, SEM. (*P < 0.05; **P < 0.01; ^§^*P* < 0.001; NS, not significant, *P* > 0.05).

To identify the role of SK1 in RAD001-induced sensitization to docetaxel, we have tested whether SK1 overexpression would reverse PC-3 cell viability and caspases activation induced by these drugs. We have used PC-3 cells stably transfected with human SK1 (PC-3/SK1, as described previously) [20, 22, 29]. PC-3/SK1 cells had 7- and 10-fold increase in SK1 activity and expression, respectively, in comparison to empty vector-transfected cells (PC-3/Neo) (Figure 3A). PC-3/Neo had similar levels of SK1 expression and activity as PC-3 wild type cells (Figure 3A, wild type was taken as 100%) and exhibited the same response to RAD001 and docetaxel as PC-3 wild type cells (PC-3/WT) (Figure 3).

**Figure 4:**
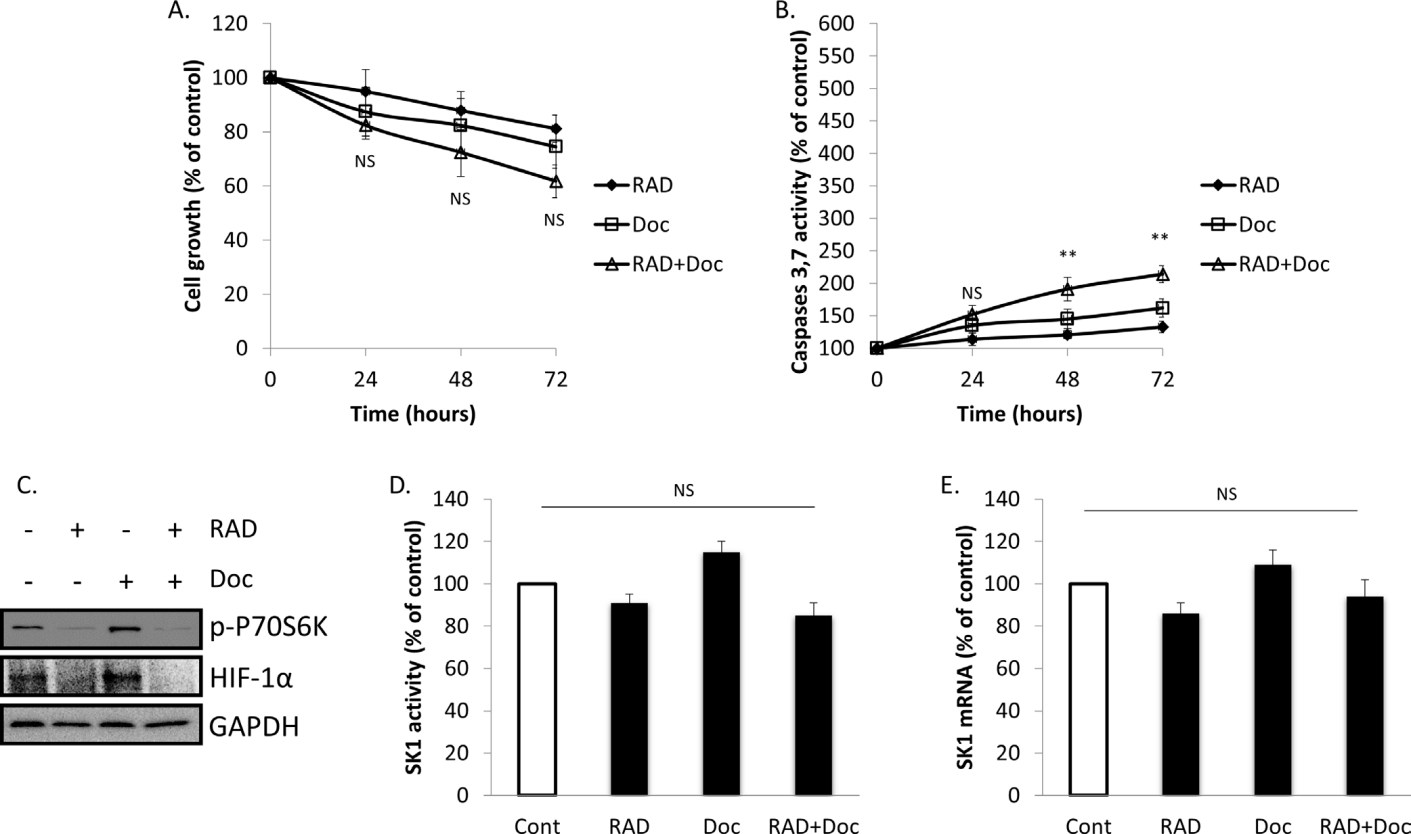
Overexpression of SK1 restores prostate cancer cells chemoresistance PC-3/SK1 cells were starved overnight then incubated with 0.1% DMSO (Cont), 100 nM RAD001 (RAD), 5 nM docetaxel (Doc) and the combination of these drugs (RAD+Doc) for 72 h (**A, B**) or 24 h (**C–E**). A. Cell proliferation was measured using MTT assay. B. Activity of caspases 3,7 was measured using caspases assay. C. Cell extracts were loaded on acrylamide mini gel and probed for p-P70S6K, HIF-1α and GAPDH. D. SK1 activity was measured using radiolabeling. E. SK1 expression was determined by qRT-PCR, normalized against housekeeping genes (GAPDH, YWHAZ and UBC) and analyzed using qBase software. Columns, mean of three independent experiments performed in triplicate; bars, SEM. (*P < 0.05; **P < 0.01; ^§^*P* < 0.001; NS, not significant, *P* > 0.05).

PC-3/SK1 cells had significant resistance to docetaxel +/– RAD001 both in terms of cell caspases 3/7 activity (Figures 3B, 3C and 4A, 4B) in comparison to PC-3/WT and PC-3/Neo cells. This correlated to the absence of significant SK1 down-regulation by any of the therapies in PC-3/SK1 cells (Figure 4D, 4E). PC-3/SK1 treated with RAD001 alone or in combination with docetaxel showed similar reduction in p-P70S6K and HIF-1α as PC-3/Neo and PC-3/WT cells (Figures 3D; 4C) suggesting that SK1 is an ultimate factor downstream of these pathways that is responsible for chemoresistance. Of note, a small increase in p-P70S6K and HIF-1α levels was noted upon SK1 overexpression, which indicates a possible feedback amplification loop (Figure 3A). Similar data were obtained in DU145/SK1 cells (Supplementary Figure S3).

### Treatment with cobalt chloride (CoCl_2_) partially restores prostate cancer cells chemoresistance and SK1 expression reduced by RAD001

**Figure 5:**
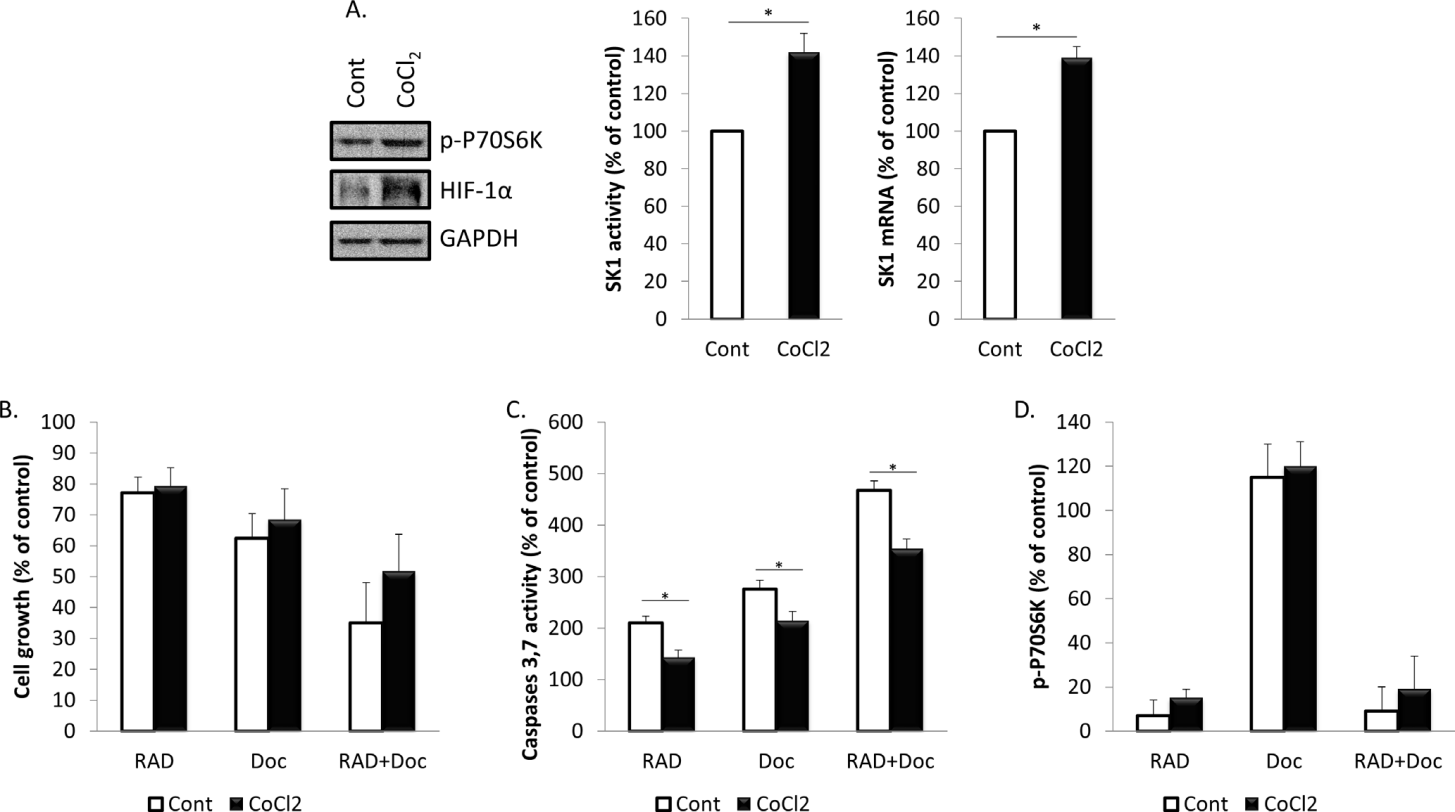
Treatment with CoCl_2_ increases SK1 expression and partially restores prostate cancer cells chemoresistance (**A**) PC-3 cells were treated with or without 100 μM of CoCl_2_ for 24 h. Cell extracts were loaded on acrylamide mini gel and probed for p-P70S6K, HIF-1α and GAPDH; SK1 activity and SK1 expression were measured using radiolabeling and qRT-PCR, respectively. (**B–D**) PC-3 cells were starved overnight, pretreated for 1 h with 100 μM of CoCl_2_ then incubated with 0.1% DMSO (Control), 100 nM RAD001 (RAD), 5 nM docetaxel (Doc) and the combination of these drugs (RAD+Doc) for 24–72 h. B. Cell proliferation was measured using MTT assay at 72 h. C. Activity of caspases 3,7 was measured using caspases assay at 72 h. D. P70S6K phosphorylation was measured using ELISA at 24 h. Data (B-D) is expressed as percent relative to relevant untreated controls. Columns, mean of three independent experiments performed in triplicate; bars, SEM. (*P < 0.05; **P < 0.01; ^§^*P* < 0.001; NS, not significant, *P* > 0.05).

**Figure 6:**
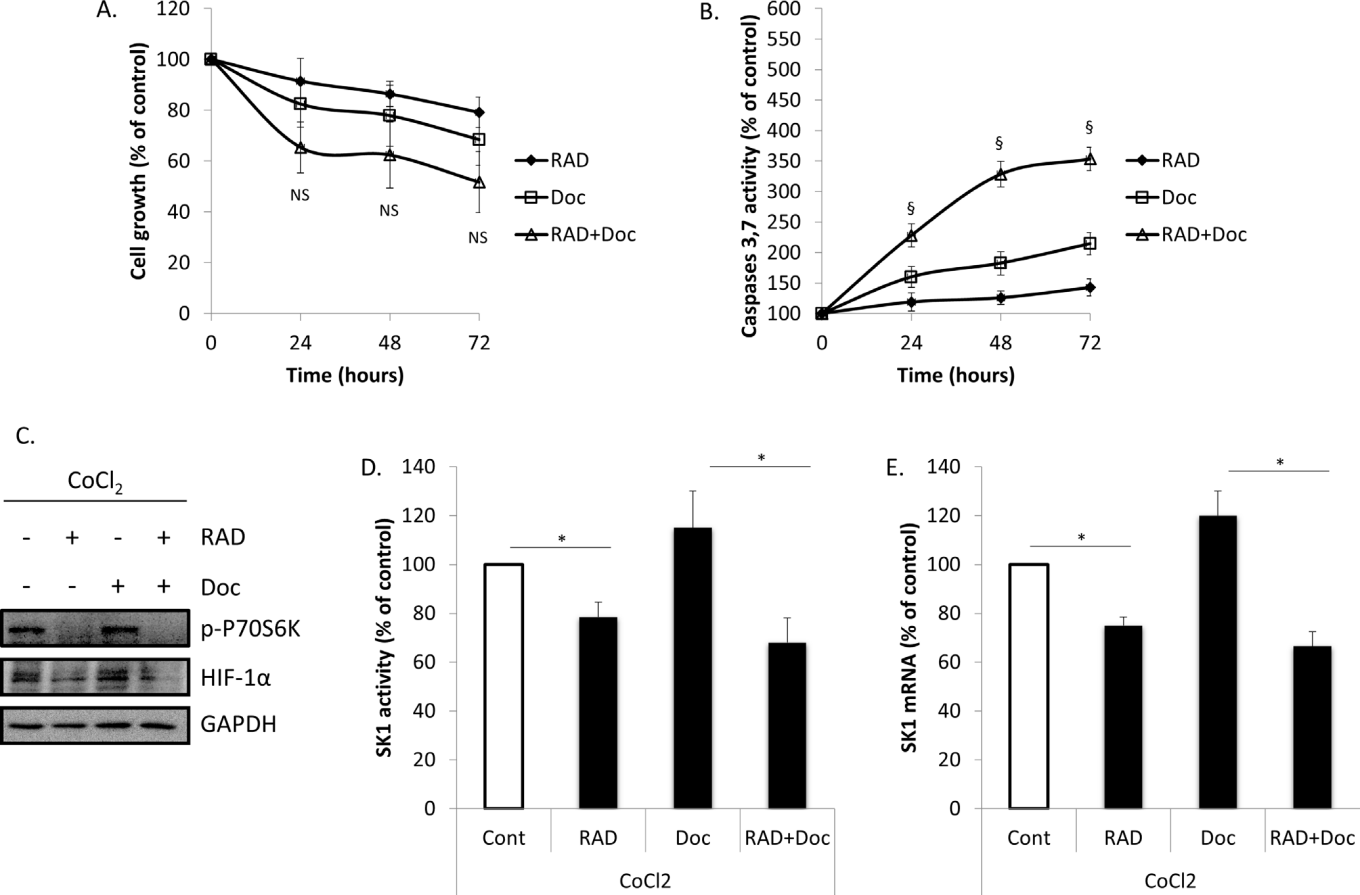
Treatment with CoCl_2_ increases HIF-1α levels, SK1 expression and prostate cancer cells chemoresistance PC-3 cells were pretreated for 1 h with 100 μM of CoCl_2_ then treated with 0.1% DMSO (Cont), 100 nM RAD001 (RAD), 5 nM docetaxel (Doc) and the combination of these drugs (RAD+Doc) for 72 h (**A, B**) or 24 h (**C–E**). A. Cell proliferation was measured using MTT assay. B. Activity of caspases 3,7 was measured using caspases assay. C. Cell extracts were loaded on acrylamide mini gel and probed for p-P70S6K, HIF-1α and GAPDH. D. SK1 activity was measured using radiolabeling. E. SK1 expression was determined by qRT-PCR, normalized against housekeeping genes (GAPDH, YWHAZ and UBC) and analyzed using qBase software. Columns, mean of three independent experiments performed in triplicate; bars, SEM. (*P < 0.05; **P < 0.01; ^§^*P* < 0.001; NS, not significant, *P* > 0.05).

**Figure 7:**
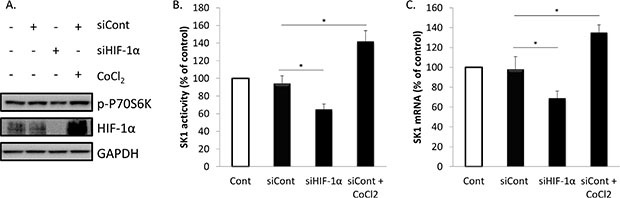
Regulation of basal SK1 expression/activity by HIF-1α PC-3 cells were transfected with specific siRNA against HIF- 1α (siHIF-1α) or control siRNA (siCont) using oligofectamine™. Cells were starved overnight in serum-free media then treated with or without 100 μM of CoCl_2_ for 24 h. (**A**) Cell extracts were loaded on acrylamide mini gel and probed for phosphorylation of P70S6K, HIF- 1α and GAPDH. (**B**) SK1 activity was measured using radiolabeling. (**C**) SK1 expression was determined by qRT-PCR, normalized against housekeeping genes (GAPDH, YWHAZ and UBC) and analyzed using qBase software. Columns, mean of three independent experiments performed in triplicate; bars, SEM. (*P < 0.05; **P < 0.01; ^§^*P* < 0.001; NS, not significant, *P* > 0.05).

We have next investigated whether HIF-1α upregulation will affect prostate cancer cell proliferation, caspases activity and SK1 expression and activity (Figures 5, 6; Supplementary Figure S4). Accumulation of HIF-1α can be induced by 100 μM CoCl_2_. Pre-treatment of PC-3 cells with CoCl_2_ increased HIF-1α protein levels, when compared with untreated cells (Figure 5A). The increase in HIF-1α protein levels was paralleled by increase in p-P70S6K levels, induction of SK1 activity by 42% and expression by 39% (Figure 5A). Pre-treatment with CoCl_2_ partially protected PC-3 cells from RAD001 and docetaxel in both cell viability and caspases activation (Figure 5B, 5C). In cells treated with CoCl_2_, 72 h combined treatment by RAD001 and docetaxel induced a 48% loss of cell viability vs 65% in untreated cells, which, however, was not significant (Figure 5B). Same treatment induced a 354% increase in caspases activity in CoCl_2_ treated cells vs 467% increase in untreated cells (Figure 5C). Like SK1 upregulation, CoCl_2_ did not abolish the effect of RAD001 alone or in combination with docetaxel on p-P70S6K (Figure 5D). Treatment with CoCl_2_ has partially restored HIF-1α levels in RAD001-treated cells (Figure 6C) and partially restored SK1 activity (Figure 6D, 6E), albeit the effect of RAD001 still remained significant. Similar outcome with regards to cell viability, caspases, p-P70S6K, HIF-1α and SK1 levels was noticed in DU145 (Supplementary Figure S4). These findings indicate that HIF-1α promotes prostate cancer cell growth and survival in the presence of chemotherapeutic agents, however the effect of its upregulation is less than that of SK1. SK1 upregulation by CoCl_2_ treatment suggests that basal SK1 levels may be under the regulation of HIF-1α pathway. To prove the possible regulation of basal SK1 expression/activity by HIF-1α, we have knocked down HIF-1α in untreated PC-3 cells using siRNA. Figure 7 shows that HIF-1α knockdown has led to a ~30% reduction in SK1 mRNA expression and enzyme activity. By contrast, CoCl_2_ has led to a 30–40% increase in SK1 expression and activity.

### RAD001 sensitizes human prostate tumors established in nude mice to docetaxel

Balbc/Nude mice were subcutaneously implanted with 10^6^ PC-3 cells and tumors were left to grow for 2 weeks. Mice were randomized into groups and treated twice a week with sham intraperitoneal injections, 5 mg/kg docetaxel, 5 mg/kg RAD001 or combination of these drugs for three weeks.

**Figure 8:**
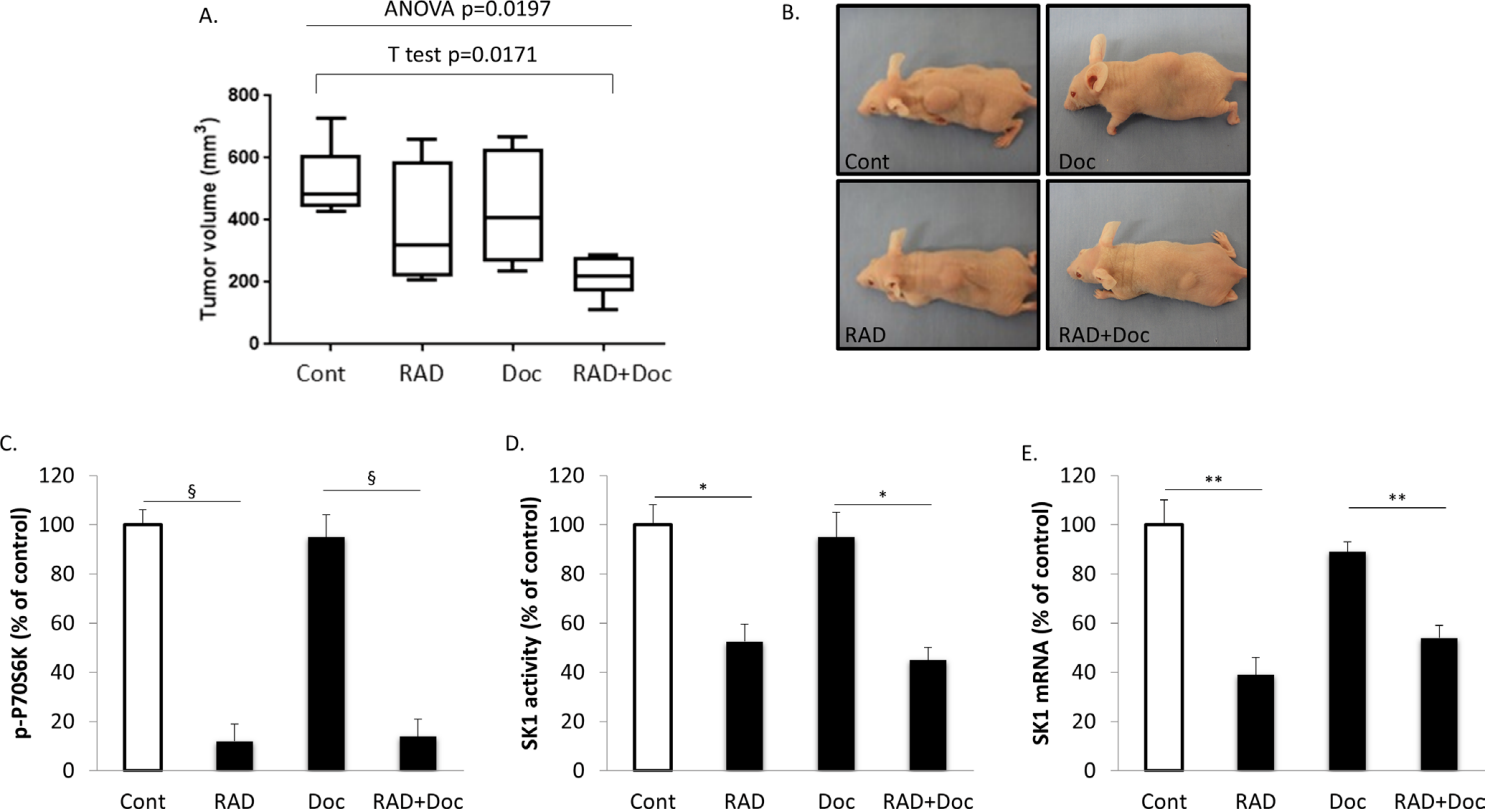
RAD001 sensitizes human prostate tumors established in nude mice to docetaxel Subcutaneous PC-3 tumors were established in nude mice as described in materials and methods and allowed to grow for two weeks. Mice were treated with sham (Cont), 5 mg/kg RAD001 (RAD), 5 mg/kg docetaxel (Doc) and combination of these drugs (RAD+Doc) for three weeks. (**A)** Tumor volumes at week five**. (B)** Representative images of subcutaneous human tumors in mice after corresponding treatments. **(C)** P70S6K phosphorylation in tumors was measured using ELISA. (**D)** SK1 activity was measured using radiolabeling. (**E)** SK1 expression was determined by qRT-PCR, normalized against housekeeping genes (GAPDH, YWHAZ and UBC) and analyzed using qBase software. Boxes, first quartile, median, second quartile; whiskers, samples falling outside upper and lower quartiles; columns, mean values of *n* = 8; bars, SEM. (*P < 0.05; **P < 0.01; ^§^*P* < 0.001; NS, not significant, *P* > 0.05).

After five weeks, subcutaneous tumors in non-treated animals reached 518±48 mm^3^, in animals treated with RAD001 alone - 397±65 mm^3^, in animals treated with docetaxel alone - 439±59 mm^3^ and in animals treated with combination therapy 218±24 mm^3^ (p=0.0171 combination treatment *vs* control; p=0.0197 overall 4 groups ANOVA) (Figure 8A, 8B).

Figure 8C, 8D, 8E shows that both RAD001 and combined therapy have significantly down-regulated tumor p-P70S6K, SK1 activity and expression. This decrease in mTOR/SK1 signaling correlated with significant (~twofold) tumor size reduction in the combination group in comparison to the single treatment groups.

**Figure 9:**
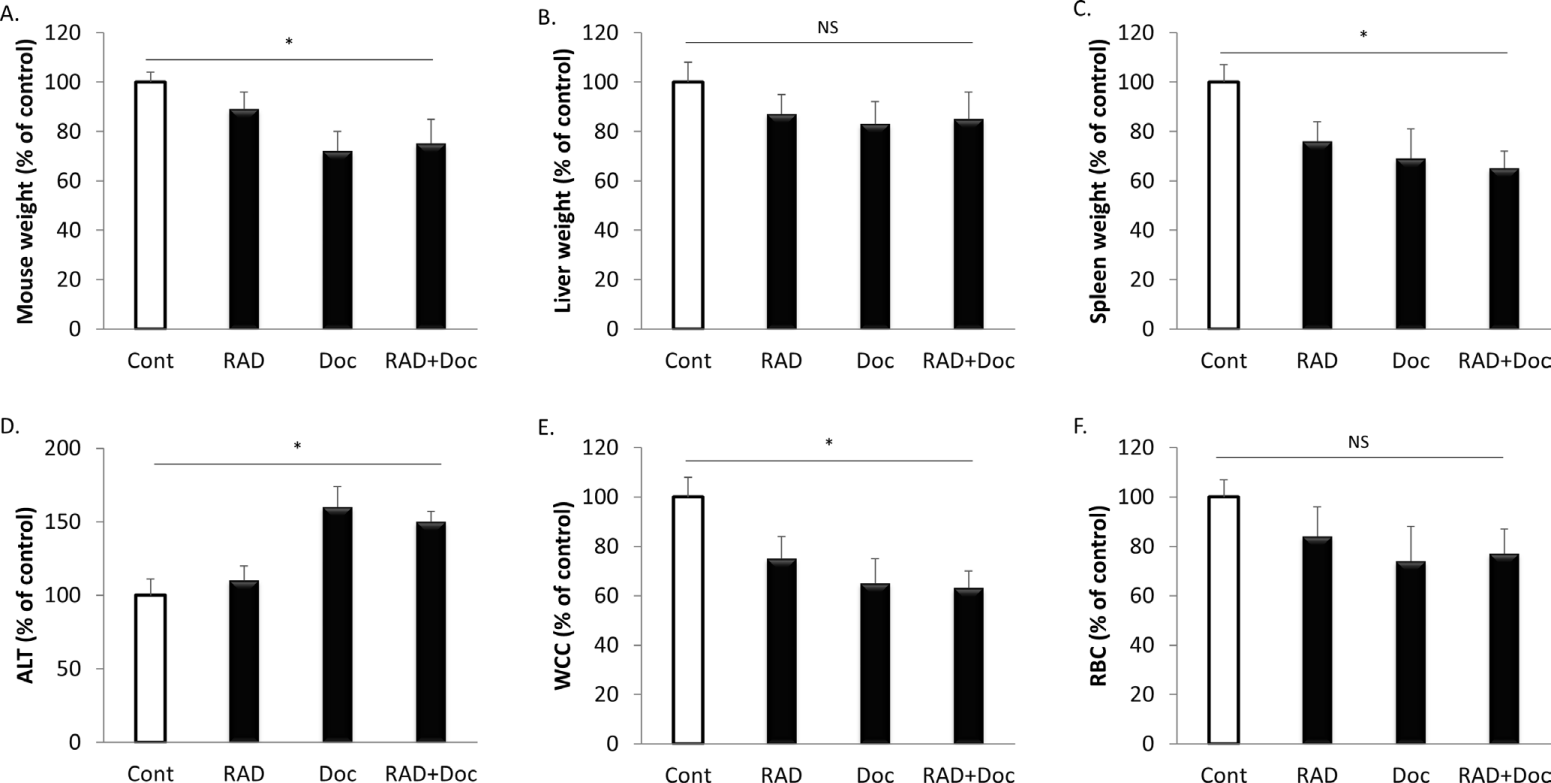
Combination of RAD001 and docetaxel has similar body toxicity as docetaxel alone Subcutaneous PC-3 tumors were established in nude mice as described in materials and methods and allowed to grow for two weeks. Mice were treated with sham (Cont), 5 mg/kg RAD001 (RAD), 5 mg/kg docetaxel (Doc) and combination of these drugs (RAD+Doc) for three weeks. All values were expressed in comparison to untreated controls taken as 100%. **(A)** Mouse total body weight. (**B)** Liver weight. (**C)** Spleen weight. (**D)** Liver alanine transaminase (ALT). (**E)** White cell count (WCC). (**F)** Red blood cell count (RBC). Columns, mean values of *n* = 8; bars, SEM. (*P < 0.05; **P < 0.01; ^§^*P* < 0.001; NS, not significant, *P* > 0.05).

We have further investigated the effects of the used treatments on mouse systemic toxicity. All treatments have reduced mouse total body weight in comparison to untreated controls (Figure 9A). Docetaxel alone was most detrimental in reducing mouse weight by 28%, while RAD001 effects were milder (11% reduction). Combined treatment was comparable to docetaxel in reducing the total body weight by 25% (Figure 9A). Liver and spleen weights were used as surrogate markers for chemotherapy-induced organ damage (Figure 9B, 9C). All therapies have reduced organ weights, and combination therapy had similar toxicity as docetaxel alone, while being twice more efficient in tumor size reduction (Figure 8A, 8B). RAD001 had a very mild liver toxicity increasing liver alanine transaminase (ALT) by 10% (not significant, Figure 9D). In contract, docetaxel has caused a 60% increase in mouse serum ALT, and the combination therapy had the same effect. All therapies have reduced white and red cell counts (Figure 9E, 9F). Likewise to other toxicity markers, combination therapy had a similar toxicity profile to docetaxel (Figure 9), while possessing its double antitumor efficacy (Figure 8).

## DISCUSSION

Docetaxel chemoresistance is an important clinical issue in prostate cancer management given that half of patients do not respond to therapy, and those who initially respond will ultimately relapse [5]. Overcoming resistance to docetaxel therapy and improving treatment outcome is a high priority and has been a challenge since docetaxel was first established as front-line therapy for mCRPC [5].

We have previously shown that SK1 mediates prostate cancer docetaxel chemoresistance [20] and we have also shown that cytotoxic concentrations of docetaxel (20 nM) inhibit SK1 in prostate cancer cells, while lower, less effective concentrations of docetaxel (5 nM) do not have such effect. We have hypothesized that this absence of SK1 inhibition could be a possible contributor to chemoresistance and proven this to be true demonstrating synergy between docetaxel and SK1 inhibitors in prostate cancer cells [22].

Several studies indicate that mTOR inhibition is a valid strategy for docetaxel sensitization in prostate cancer. Wu *et al* have demonstrated that mTOR inhibitor CCI-779 (temsirolimus) can sensitize prostate cancer cells to docetaxel [11], while Morgan *et al* have shown that RAD001 and docetaxel produce synergy in blocking formation of prostate bone metastases [18]. One mechanistic study has suggested that mTOR inhibitor-induced chemosensitization may be mediated by survivin down-regulation [30]. Zhou *et al* have shown that nanoparticles containing mTOR inhibitor and docetaxel suppressed prostate stem/progenitor cell growth [31].

Here we show for the first time that in prostate cancer cells mTORC1 increases SK1 expression through HIF-1α in normoxic conditions, a pathway that may be interrupted by mTOR inhibitor RAD001. Zhang *et al* suggested that WYE-132, an mTORC1/2 dual inhibitor, may target SK1 independently from mTOR inhibition, but no clear mechanism was provided [32]. Our *in situ* kinetic studies did not show that RAD001 is a direct SK1 inhibitor (data not shown). In other systems, SK1 expression was shown to be induced by mTOR kinase [23, 33]. Indeed, Figure 2 and Supplementary Figure S2 show that in normoxic conditions RAD001 blocks mTORC1 (assessed via p-P70S6K) and down-regulates HIF-1α expression, SK1 expression and activity. This is the first evidence of mTOR-mediated regulation of HIF-1α in prostate cancer. Of interest RAD001 did not only decrease basal HIF-1α levels, but also those induced by CoCl_2_, indicating that HIF-1α biosynthesis depends on mTOR. This is supported by previous studies proposing that in other systems the expression of HIF-1α is under mTOR regulation [24]. On the contrary, in non-cancer epithelial cells rapamycin only marginally inhibited desferrioxamine-induced HIF-1α and transcription [34]. HIF-1α, in turn increases synthesis of growth factors, which further activate mTOR (reviewed in [34]). The clinical importance of our findings is supported by data showing increased HIF-1α levels in primary prostate cancer and its key role in disease progression [35]. Recent findings suggest that HIF-1α may also increase prostate cancer chemoresistance [36]. Interestingly, it has been reported that HIF-1α may in turn stimulate mTOR signaling in prostate cancer stem cells leading to their resistance to selective mTOR inhibitors [37]. In concordance with that, we observed an increase in P70S6K phosphorylation upon HIF-1α induction by CoCl_2_ (Figure 5A; Supplementary Figure S4A). This clearly indicates the presence of a feedback loop from HIF-1α to mTOR signaling, which may promote cancer cell survival in the hypoxic niche [37]. HIF-1α might lead to an increase in mTOR activity by reducing intracellular adenosine monophosphate (AMP) levels, thus deactivating AMP kinase, which normally blocks mTOR activity by phosphorylating it at T2446 [38]. It therefore makes it harder to discern the influence of the individual loop components on the downstream targets.

In a knockdown experiment we show that HIF- 1α stimulates SK1 expression during normoxia as HIF- 1α siRNA down-regulates SK1 and CoCl_2_ increases its expression (Figure 7). HIF-1α was previously shown to increase SK1 expression in other systems [25, 26]. Also, an opposite effect was shown where SK1 was demonstrated to increase HIF-1α stability [39]. We have indeed observed that SK1 overexpression leads to upregulated levels of HIF-1α and p-P70S6K (Figure 3A; Supplementary Figure S3A), suggesting a positive feedback loop mechanism (possibly through S1P receptors [39, 40]), which we, however did not investigate as this was not the primary purpose of this publication. Importantly, SK1 overexpression protects prostate cancer cells from RAD001 and docetaxel treatment (Figures 3, 4; Supplementary Figure S3). Our previous data suggest that sustained SK1 activity increases prostate cancer cell chemoresistance [22]. This protection persists during RAD001-mediated reduction of HIF-1α and mTOR signaling (Figures 3, 4; Supplementary Figure S3), suggesting SK1 being an ultimate downstream target of these pathways. In contrast to SK1 overexpression, a significant upregulation of HIF-1α by CoCl_2_ had a proportionally smaller effect on both SK1 expression and restoring cell viability (Figures 5, 6, Supplementary Figure S4). We have shown that SK1 activity is increased in human prostate tumors and correlated with poor prognosis [41]. This highlights the clear importance of SK1 as a signaling hub and a therapy target in prostate cancer.

Here we show that SK1 down-regulation by RAD001 allows chemosensitization to 5 nM docetaxel (Figure 1, Supplementary Figure S1), while its enforced expression protects cancer cells from chemotherapy induced cell death (Figures 3, 4, Supplementary Figure S3). This finding is of clear translational importance as previously we have shown that 20 nM docetaxel is required to successfully induce apoptosis in PC-3 cells as a single therapy [22], therefore, a 5 nM dose represents a significant (4-fold) reduction in the effective dose suggesting RAD001 as a potent sensitizer to low doses of docetaxel in prostate cancer cell culture models.

Our mouse study showed a significant advantage of using docetaxel in combination with RAD001. Both therapies worked in synergy leading to tumor size reduction (Figure 8). Similar to *in vitro* studies – they caused down-regulation of p-P70S6K, SK1 activity and expression. Importantly the toxicity of combination therapy never exceeded the toxicities of individual therapies, indicating a significant advantage of using this drug combination (Figure 9).

Our *in vivo* toxicity data is supported by a phase I study of RAD001 and docetaxel in patients with mCRPC, which found that RAD001 10 mg daily and docetaxel 60 mg/m^2^ was safe in mCRPC patients [19]. Another phase II study has shown that another mTOR inhibitor temsirolimus may work as a successful maintenance therapy after docetaxel induction and does not adversely affect quality of life [42]. While in this study the evidence of using combination therapy is indirect (both therapies were not administered together), it encourages further investigation of mTOR inhibitors in prostate cancer.

**Figure 10:**
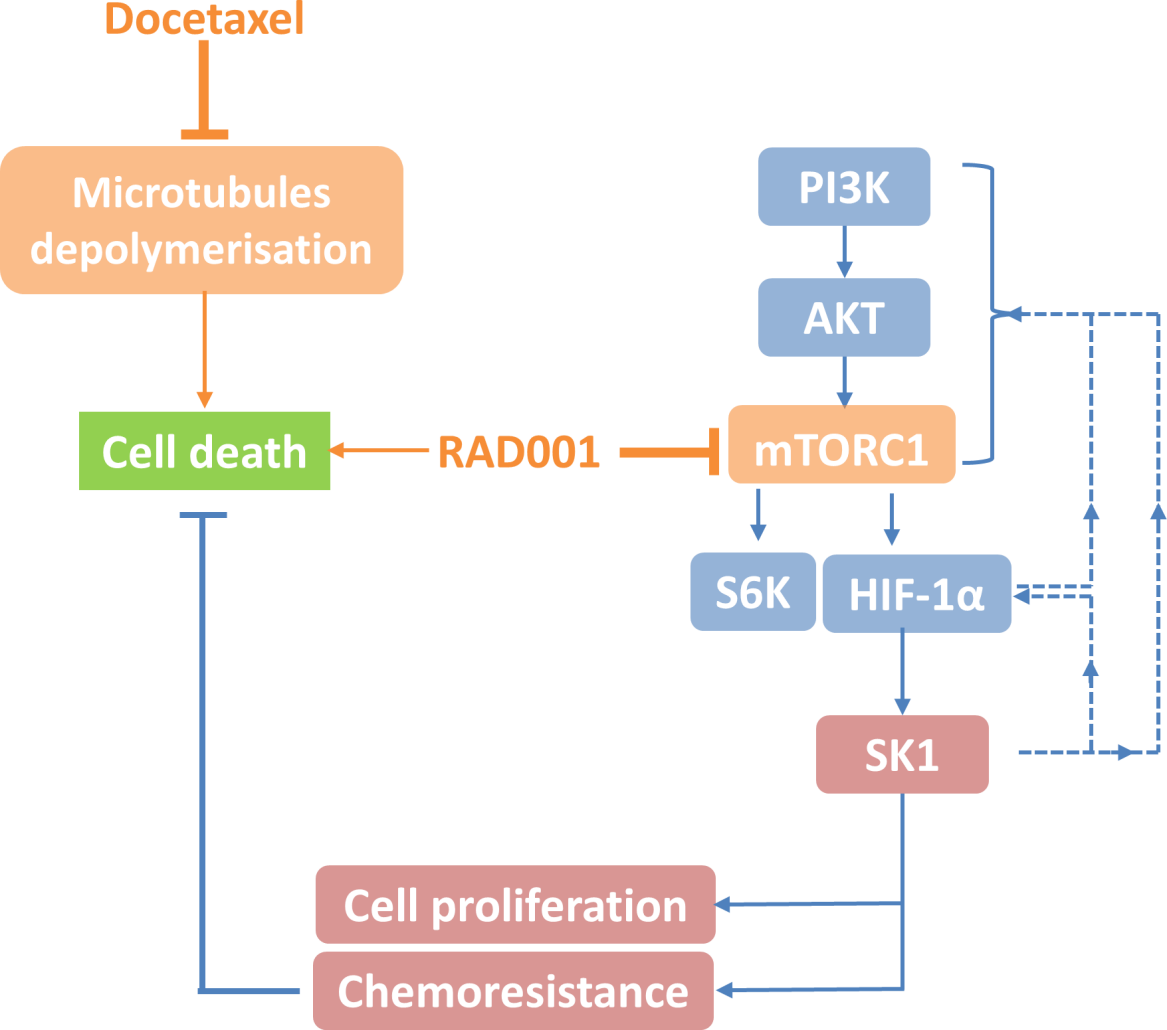
Schematic representation of proposed mTOR/HIF-1α/SK1 pathway and its role in docetaxel chemosensitization in prostate cancer As previously described docetaxel inhibits microtubule depolymerization and induces cancer cell death while sustained SK1 activity/expression contributes to cancer cell proliferation and chemoresistance [20–22]. RAD001 inhibits mammalian target of rapamycin (mTOR) complex 1 (mTORC1) and down-regulates hypoxia induced factor-1α (HIF-1α) and sphingosine kinase 1 (SK1). SK1 overexpression can block RAD001 effects. SK1 overexpression also induces partial reactivation of mTOR/HIF-1α pathway, suggesting a positive feedback loop mechanism (dashed line, not investigated in this study). Solid lines – mechanisms investigated in this study. Dashed lines - possible feedback pathways. Phosphoinositide 3-kinase (PI3K).

The major findings of this work are summarized in Table 1. Overall, in our paper, we provide the first mechanistic link between mTOR inhibition and docetaxel resistance in prostate cancer implicating the regulation of HIF-1α/SK1 pathways (Figure 10). In comparison to previous studies, our data show that RAD001 allows a 4-fold reduction of effective docetaxel dose both *in vitro* and *in vivo* and that the toxicity of combined therapy does not exceed that of docetaxel chemotherapy alone. Our findings suggest that combinational therapies that target mTOR/HIF-1α/SK1 may overcome docetaxel resistance and have a clinical benefit in human cancers. Both RAD001 and docetaxel are clinically approved and safety early phase trials indicate feasibility of their use in prostate cancer patients. RAD001 is now extensively tested in breast cancer [43] and more clinical trials are required in prostate cancer.

**Table 1:** List of the major findings

1.	mTOR inhibition sensitizes prostate cancer cells to small dose of docetaxel
2.	mTOR inhibition downregulates the levels of HIF-1α
3.	mTOR inhibition decreases SK1 expression
4.	HIF-1α promotes SK1 expression, but also increases mTOR activity (positive feedback loop)
5.	SK1 overexpression abrogates the effect of mTOR inhibition on prostate cancer cell viability
6.	SK1 overexpression induces an increase in mTOR activity and HIF-1α protein content (second positive feedback loop)
7.	mTOR inhibition synergizes with docetaxel to induce a significant reduction of human prostate tumors in mice
8.	The toxicity of combined therapy does not exceed the toxicity of single treatments

## MATERIALS AND METHODS

### Cell lines and cell culture

Androgen insensitive prostate cancer cell lines (PC-3 and DU145) were obtained from DSMZ (Braunschweig, Germany). PC-3/SK1 and DU145/SK1 were derived from parental cell lines through stable transfection with human SK1 [44]. Cells were maintained in tissue culture flasks or plastic dishes in a humidified atmosphere of 5% CO_2_ at 37°C using Roswell Park Memorial Institute (RPMI) 1640 supplemented with 10% heat-inactivated fetal bovine serum (FBS) (Sigma-Aldrich, UK), 50 U/ml penicillin, 50 μg/ml streptomycin and 2 mM glutamine (Sigma-Aldrich, UK). In the case of stably transfected prostate cancer cell lines, the growth medium was supplemented with 1 mg/ ml Geneticin sulfate (G418, Santa Cruz Biotechnology, Heidelberg, Germany). Cell lines were routinely verified by morphology and growth curve analysis and routinely screened for mycoplasma infection (using MP0035 Lookout, Sigma). All experiments were conducted in the absence of serum. Cells were seeded to be 80% confluent by the end of treatment and were treated as indicated in figures' legends. Cell lines were kept in culture for up to 30 passages.

### Reagents

Silica gel 60 high-performance TLC plates were from VWR (West Chester, PA, USA), and [γ-^32^P]-ATP was purchased from Perkin-Elmer (Waltham, MA, USA). Everolimus (RAD001) from Selleckchem (Newmarket, UK). All other chemicals were from Sigma Aldrich (Poole, UK).

### Cell viability

Cells were grown in 96-well plates, starved, and exposed to different treatments as indicated in figure legends. Cellular viability was measured using the 3-(4,5-dimethylthiazol-2-yl)-2,5-diphenyltetrazolium bromide (MTT; 5 mg/ml) colorimetric assay as already described [45].

### Caspase assay

Caspase assay was performed in 96-well plates using caspases-3,7-Glo substrate (Promega, Madison, WI, USA) according to the manufacturer's instructions as described before [22].

### Cell treatment and preparation of cell lysates

Cells were plated, serum deprived, and treated as indicated in figure legends. After incubation, cells were washed with ice-cold phosphate-buffered saline (PBS) and harvested.

### Western blotting

The levels of HIF-1α (Abcam; Cambridge, UK), p-P70S6K(Thr389) (New England Biolabs; Hitchin, UK), and glyceraldehyde-3-phosphate dehydrogenase (GAPDH) (Santa Cruz Biotechnology, Heidelberg, Germany) were determined by Western blot analysis as previously described [45, 46]. Following treatment and incubation for 24 h cells were collected as described above. Cell extracts were loaded on acrylamide mini gels and separated proteins were transferred onto PVDF Immobilon-P^®^ membranes then blocked in PBS-Tween containing 5% (w/v) non-fat dry milk. Primary antibodies were incubated overnight at 4°C. Secondary peroxidase-conjugated antibodies (GE healthcare, Buckinghamshire, UK) were added in PBS-Tween/milk. Membranes were exposed to chemiluminescent HRP substrate (GE Healthcare Buckinghamshire, UK) and visualised using the ChemiDoc-IT Imaging System (UVP, Bio-Rad, Hertfordshire, UK).

### ELISA for p-P70S6K (Thr389)

For the measurements of p-P70S6K, cell lysates were prepared and ELISA was conducted as previously described [47]. Equal amount of lysates was loaded onto PathScan p-P70S6K plates and PathScan total P70S6K plates (Cell Signaling, Danvers, USA), and the assay was performed according to manufacturer's instructions. Levels of p-P70S6K were normalized to corresponding total P70S6K levels.

### RNA extraction and cDNA synthesis and qRT-PCR

Isolation of total RNA from prostate cancer cell lines and mouse tumors was performed using the RNeasy Mini kit (Qiagen, Valencia, CA, USA) as per manufacturer's instructions. RNA quantity and purity was measured using a NanoDrop ND-100 Spectrophotometer (Thermo Fisher Scientific, Loughborough, UK). Reverse transcription was performed using Precision nanoScript™ Reverse transcription kit (PrimerDesign Ltd, Southampton, UK). qRT-PCR was done as already described [45]. The expression of SK1 was normalized to three reference genes: ubiquitin C (UBC), GAPDH and tyrosine-3-monooxy-genase/tryptophan 5-mono-oxygenase activation protein (YWAZH). Ct values were exported and analyzed using qbase software (Biogazelle NV, Zwijnaarde, Belgium) using multiple reference genes normalization.

### Sphingosine kinase 1 assay

SK1 assay was performed using radiolabeling as previously described [46, 48], in conditions favoring SK1 activity and inhibiting SK2 activity.

### RNA interference

Cells were seeded at a density to reach 30–50% confluence by the day of transfection. Cells were transfected as described previously [45] with small interfering RNA (siRNA) against HIF-1α (siHIF-1α) (Ambion/Life Technologies) using Oligofectamine (Invitrogen, Carlsbad, CA) according to the manufacturer's instructions. Non-targeting siRNA, were used as a negative control. Optimal knockdown was verified by western blot.

### Animal study

Animal studies were conducted under Home Office license and were approved by the Institutional Animal Care and Use Committee. Animal study was performed as previously described [20, 29]. Briefly, subcutaneous human prostate cancer xenografts were established in BALBc/nude male mice by subcutaneous injection of 1*10^6^ PC-3 cells. Two weeks after implantation, mice were randomized into treatment groups and treated twice a week for three weeks with: i.p. injections of vehicle (control), 5 mg/kg docetaxel, 5 mg/kg RAD001 and a combination of these drugs. One day after the last treatment, all mice were euthanized. Tumor long and short radii were measured using calipers and tumor volume (v) was calculated using the formula v = 4/3 πab^2^ (a – long radius, b – short radius). Mice and individual organs were weighed and primary tumors were then processed for analysis of SK1 activity, expression and p-P70S6K quantification as described above. Blood counts and alanine aminotransferase (ALT) were done in the Hammersmith hospital biochemical lab.

### Statistical analysis

Data are presented as the mean values of at least three independent experiments normalized to control ± standard error of the mean (SEM) calculated using GraphPad Prism. Statistical significance between two groups was conducted by unpaired Student's *t* test. Comparisons between the means of more than two groups were assessed using one-way ANOVA analysis followed by a Tukey's test (95 % confidence). *P* value of < 0.05 is considered statistically significant.

## SUPPLEMENTARY MATERIALS



## References

[1] Torre LA, Bray F, Siegel RL, Ferlay J, Lortet-Tieulent J, Jemal A (2015). Global cancer statistics, 2012. CA Cancer J Clin.

[2] Nelson WG, De Marzo AM, Isaacs WB (2003). Prostate cancer. N Engl J Med.

[3] Watson PA, Arora VK, Sawyers CL (2015). Emerging mechanisms of resistance to androgen receptor inhibitors in prostate cancer. Nat Rev Cancer.

[4] Steineck G, Reuter V, Kelly WK, Frank R, Schwartz L, Scher HI (2002). Cytotoxic treatment of aggressive prostate tumors with or without neuroendocrine elements. Acta Oncol.

[5] Tannock IF, de Wit R, Berry WR, Horti J, Pluzanska A, Chi KN, Oudard S, Theodore C, James ND, Turesson I, Rosenthal MA, Eisenberger MA (2004). Docetaxel plus prednisone or mitoxantrone plus prednisone for advanced prostate cancer. N Engl J Med.

[6] Gravis G, Fizazi K, Joly F, Oudard S, Priou F, Esterni B, Latorzeff I, Delva R, Krakowski I, Laguerre B, Rolland F, Theodore C, Deplanque G (2013). Androgen-deprivation therapy alone or with docetaxel in non-castrate metastatic prostate cancer (GETUG-AFU 15): a randomised, open-label, phase 3 trial. Lancet Oncol.

[7] James ND, Sydes MR, Clarke NW, Mason MD, Dearnaley DP, Spears MR, Ritchie AWS, Parker CC, Russell JM, Attard G, de Bono J, Cross W, Jones RJ Addition of docetaxel, zoledronic acid, or both to first-line long-term hormone therapy in prostate cancer (STAMPEDE): survival results from an adaptive, multiarm, multistage, platform randomised controlled trial. Lancet.

[8] McMenamin ME, Soung P, Perera S, Kaplan I, Loda M, Sellers WR (1999). Loss of PTEN Expression in Paraffin-embedded Primary Prostate Cancer Correlates with High Gleason Score and Advanced Stage. Cancer Res.

[9] Bitting RL, Armstrong AJ (2013). Targeting the PI3K/Akt/mTOR pathway in castration-resistant prostate cancer. Endocr Relat Cancer.

[10] Schwartz S, Wongvipat J, Trigwell CB, Hancox U, Carver BS, Rodrik-Outmezguine V, Will M, Yellen P, de Stanchina E, Baselga J, Scher HI, Barry ST, Sawyers CL (2015). Feedback suppression of PI3Kalpha signaling in PTEN-mutated tumors is relieved by selective inhibition of PI3Kbeta. Cancer Cell.

[11] Wu L, Birle DC, Tannock IF (2005). Effects of the mammalian target of rapamycin inhibitor CCI-779 used alone or with chemotherapy on human prostate cancer cells and xenografts. Cancer Res.

[12] Dancey J (2010). mTOR signaling and drug development in cancer. Nat Rev Clin Oncol.

[13] Motzer RJ, Escudier B, Oudard S, Hutson TE, Porta C, Bracarda S, Grunwald V, Thompson JA, Figlin RA, Hollaender N, Urbanowitz G, Berg WJ, Kay A (2008). Efficacy of everolimus in advanced renal cell carcinoma: a double-blind, randomised, placebo-controlled phase III trial. Lancet.

[14] Majumder PK, Febbo PG, Bikoff R, Berger R, Xue Q, McMahon LM, Manola J, Brugarolas J, McDonnell TJ, Golub TR, Loda M, Lane HA, Sellers WR (2004). mTOR inhibition reverses Akt-dependent prostate intraepithelial neoplasia through regulation of apoptotic and HIF-1-dependent pathways. Nat Med.

[15] Templeton AJ, Dutoit V, Cathomas R, Rothermundt C, Bartschi D, Droge C, Gautschi O, Borner M, Fechter E, Stenner F, Winterhalder R, Muller B, Schiess R (2013). Phase 2 trial of single-agent everolimus in chemotherapy-naive patients with castration-resistant prostate cancer (SAKK 08/08). Eur Urol.

[16] Armstrong AJ, Shen T, Halabi S, Kemeny G, Bitting RL, Kartcheske P, Embree E, Morris K, Winters C, Jaffe T, Fleming M, George DJ (2013). A phase II trial of temsirolimus in men with castration-resistant metastatic prostate cancer. Clin Genitourin Cancer.

[17] Amato RJ, Jac J, Mohammad T, Saxena S (2008). Pilot study of rapamycin in patients with hormone-refractory prostate cancer. Clin Genitourin Cancer.

[18] Morgan TM, Pitts TE, Gross TS, Poliachik SL, Vessella RL, Corey E (2008). RAD001 (Everolimus) inhibits growth of prostate cancer in the bone and the inhibitory effects are increased by combination with docetaxel and zoledronic acid. Prostate.

[19] Courtney KD, Manola JB, Elfiky AA, Ross R, Oh WK, Yap JT, Van den Abbeele AD, Ryan CW, Beer TM, Loda M, Priolo C, Kantoff P, Taplin ME (2015). A phase I study of everolimus and docetaxel in patients with castration-resistant prostate cancer. Clin Genitourin Cancer.

[20] Pchejetski D, Golzio M, Bonhoure E, Calvet C, Doumerc N, Garcia V, Mazerolles C, Rischmann P, Teissie J, Malavaud B, Cuvillier O (2005). Sphingosine kinase-1 as a chemotherapy sensor in prostate adenocarcinoma cell and mouse models. Cancer Res.

[21] Pchejetski D, Doumerc N, Golzio M, Naymark M, Teissie J, Kohama T, Waxman J, Malavaud B, Cuvillier O (2008). Chemosensitizing effects of sphingosine kinase-1 inhibition in prostate cancer cell and animal models. Mol Cancer Ther.

[22] Sauer L, Nunes J, Salunkhe V, Skalska L, Kohama T, Cuvillier O, Waxman J, Pchejetski D (2009). Sphingosine kinase 1 inhibition sensitizes hormone-resistant prostate cancer to docetaxel. Int J Cancer.

[23] Marfe G, Di Stefano C, Gambacurta A, Ottone T, Martini V, Abruzzese E, Mologni L, Sinibaldi-Salimei P, de Fabritis P, Gambacorti-Passerini C, Amadori S, Birge RB (2011). Sphingosine kinase 1 overexpression is regulated by signaling through PI3K, AKT2, and mTOR in imatinib-resistant chronic myeloid leukemia cells. Exp Hematol.

[24] Hudson CC, Liu M, Chiang GG, Otterness DM, Loomis DC, Kaper F, Giaccia AJ, Abraham RT (2002). Regulation of hypoxia-inducible factor 1alpha expression and function by the mammalian target of rapamycin. Mol Cell Biol.

[25] Lee HT, Park SW, Kim M, Ham A, Anderson LJ, Brown KM, D'Agati VD, Cox GN (2012). Interleukin-11 protects against renal ischemia and reperfusion injury. Am J Physiol Renal Physiol.

[26] Schwalm S, Doll F, Romer I, Bubnova S, Pfeilschifter J, Huwiler A (2008). Sphingosine kinase-1 is a hypoxia-regulated gene that stimulates migration of human endothelial cells. Biochem Biophys Res Commun.

[27] Burnett PE, Barrow RK, Cohen NA, Snyder SH, Sabatini DM (1998). RAFT1 phosphorylation of the translational regulators p70 S6 kinase and 4E-BP1. Proc Natl Acad Sci U S A.

[28] Saran U, Foti M, Dufour JF (2015). Cellular and molecular effects of the mTOR inhibitor everolimus. Clinical science (London, England : 1979).

[29] Pchejetski D, Bohler T, Brizuela L, Sauer L, Doumerc N, Golzio M, Salunkhe V, Teissie J, Malavaud B, Waxman J, Cuvillier O (2010). FTY720 (fingolimod) sensitizes prostate cancer cells to radiotherapy by inhibition of sphingosine kinase-1. Cancer Res.

[30] Morikawa Y, Koike H, Sekine Y, Matsui H, Shibata Y, Ito K, Suzuki K (2012). Rapamycin enhances docetaxel-induced cytotoxicity in a androgen-independent prostate cancer xenograft model by survivin downregulation. Biochem Biophys Res Commun.

[31] Zhou Y, Yang J, Zhang R, Kopecek J (2015). Combination therapy of prostate cancer with HPMA copolymer conjugates containing PI3K/mTOR inhibitor and docetaxel. Eur J Pharm Biopharm.

[32] Zhang D, Xia H, Zhang W, Fang B (2016). The anti-ovarian cancer activity by WYE-132, a mTORC1/2 dual inhibitor. Tumour Biol.

[33] Francy JM, Nag A, Conroy EJ, Hengst JA, Yun JK (2007). Sphingosine kinase 1 expression is regulated by signaling through PI3K, AKT2, and mTOR in human coronary artery smooth muscle cells. Biochimica et biophysica acta.

[34] Demidenko ZN, Blagosklonny MV (2011). The purpose of the HIF-1/PHD feedback loop: to limit mTOR-induced HIF-1alpha. Cell Cycle.

[35] Zhong H, Semenza GL, Simons JW, De Marzo AM (2004). Up-regulation of hypoxia-inducible factor 1alpha is an early event in prostate carcinogenesis. Cancer Detect Prev.

[36] Ranasinghe WK, Xiao L, Kovac S, Chang M, Michiels C, Bolton D, Shulkes A, Baldwin GS, Patel O (2013). The role of hypoxia-inducible factor 1alpha in determining the properties of castrate-resistant prostate cancers. PloS one.

[37] Marhold M, Tomasich E, El-Gazzar A, Heller G, Spittler A, Horvat R, Krainer M, Horak P (2015). HIF1alpha Regulates mTOR Signaling and Viability of Prostate Cancer Stem Cells. Mol Cancer Res.

[38] Cheng SW, Fryer LG, Carling D, Shepherd PR (2004). Thr2446 is a novel mammalian target of rapamycin (mTOR) phosphorylation site regulated by nutrient status. J Biol Chem.

[39] Ader I, Brizuela L, Bouquerel P, Malavaud B, Cuvillier O (2008). Sphingosine kinase 1: a new modulator of hypoxia inducible factor 1alpha during hypoxia in human cancer cells. Cancer Res.

[40] Liu G, Yang K, Burns S, Shrestha S, Chi H (2010). The S1P(1)-mTOR axis directs the reciprocal differentiation of T(H)1 and T(reg) cells. Nat Immunol.

[41] Malavaud B, Pchejetski D, Mazerolles C, de Paiva GR, Calvet C, Doumerc N, Pitson S, Rischmann P, Cuvillier O (2010). Sphingosine kinase-1 activity and expression in human prostate cancer resection specimens. Eur J Cancer.

[42] Emmenegger U, Booth CM, Berry S, Sridhar SS, Winquist E, Bandali N, Chow A, Lee C, Xu P, Man S, Kerbel RS, Ko YJ (2015). Temsirolimus Maintenance Therapy After Docetaxel Induction in Castration-Resistant Prostate Cancer. Oncologist.

[43] Hurvitz SA, Andre F, Jiang Z, Shao Z, Mano MS, Neciosup SP, Tseng LM, Zhang Q, Shen K, Liu D, Dreosti LM, Burris HA, Toi M (2015). Combination of everolimus with trastuzumab plus paclitaxel as first-line treatment for patients with HER2-positive advanced breast cancer (BOLERO-1): a phase 3, randomised, double-blind, multicentre trial. Lancet Oncol.

[44] Pitson SM, Moretti PA, Zebol JR, Xia P, Gamble JR, Vadas MA, D'Andrea RJ, Wattenberg BW (2000). Expression of a catalytically inactive sphingosine kinase mutant blocks agonist-induced sphingosine kinase activation. A dominant-negative sphingosine kinase. J Biol Chem.

[45] Alshaker H, Krell J, Frampton AE, Waxman J, Blyuss O, Zaikin A, Winkler M, Stebbing J, Yague E, Pchejetski D (2014). Leptin induces upregulation of sphingosine kinase 1 in oestrogen receptor-negative breast cancer via Src family kinase-mediated, janus kinase 2-independent pathway. Breast Cancer Res.

[46] Pchejetski D, Nunes J, Coughlan K, Lall H, Pitson SM, Waxman J, Sumbayev VV (2011). The involvement of sphingosine kinase 1 in LPS-induced Toll-like receptor 4-mediated accumulation of HIF-1alpha protein, activation of ASK1 and production of the pro-inflammatory cytokine IL-6. Immunol Cell Biol.

[47] Ganapathy B, Nandhagopal N, Polizzotti BD, Bennett D, Asan A, Wu Y, Kuhn B (2016). Neuregulin-1 Administration Protocols Sufficient for Stimulating Cardiac Regeneration in Young Mice Do Not Induce Somatic, Organ, or Neoplastic Growth. PloS one.

[48] Alshaker H, Wang Q, Frampton AE, Krell J, Waxman J, Winkler M, Stebbing J, Cooper C, Yague E, Pchejetski D (2015). Sphingosine kinase 1 contributes to leptin-induced STAT3 phosphorylation through IL-6/gp130 transactivation in oestrogen receptor-negative breast cancer. Breast Cancer Res Treat.

