# Supplementary material for: Everolimus (RAD001) sensitizes prostate cancer cells to docetaxel by down-regulation of HIF-1α and sphingosine kinase 1
Source: Oncotarget. 2016 Nov 4;7(49):80943–56. doi: 10.18632/oncotarget.13115 (PMC5348367; doi:10.18632/oncotarget.13115)
Supplement: Supplementary file 1 [file oncotarget-07-80943-s001.pdf]

Everolimus (RAD001) sensitizes prostate cancer cells to docetaxel by down-regulation of HIF-1α and sphingosine kinase 1

Supplementary Materials

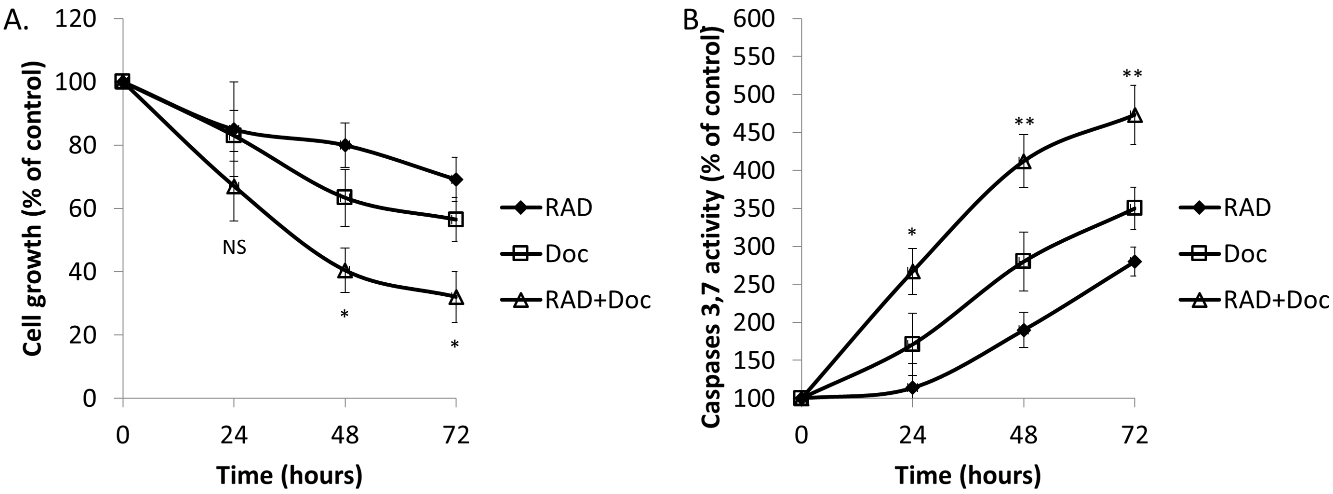

Supplementary Figure S1: RAD001 sensitises prostate cancer cells to small doses of docetaxel. DU145 cells were starved overnight then incubated with 0.1% DMSO (Cont), 100 nM RAD001 (RAD), 5 nM docetaxel (Doc) and the combination of these drugs (RAD + Doc) for 72h. (**A**) Cell proliferation was measured using MTT assay. (**B**) Activity of caspases 3,7 was measured using caspases assay. Points, mean of three independent experiments performed in triplicate; bars, SEM. ($^{*}P < 0.05$; $^{**}P < 0.01$; $^{\S}P < 0.001$; NS, not significant, $P > 0.05$).

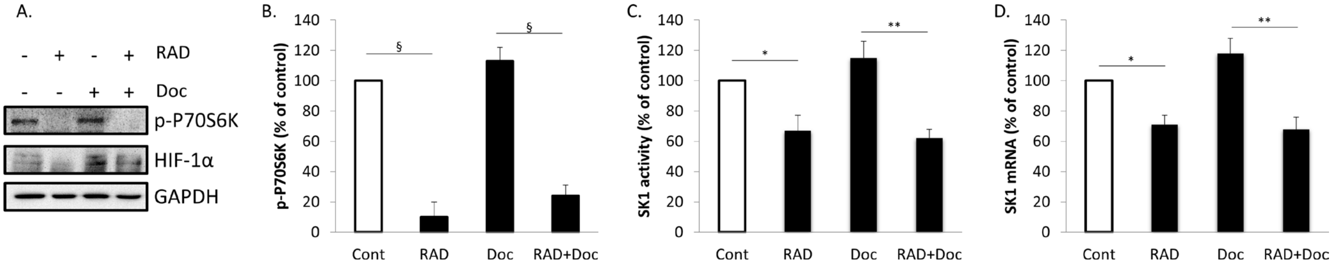

Supplementary Figure S2: RAD001 decreases P70S6K phosphorylation, HIF-1α protein levels, SK1 expression and activity. DU145 cells were starved overnight then incubated with 0.1% DMSO (Cont), 100 nM RAD001 (RAD), 5 nM docetaxel (Doc) and the combination of these drugs (RAD + Doc) for 24 h. (**A**) Cell extracts were loaded on acrylamide mini gel and probed for phosphorylation of P70S6K, HIF-1α and GAPDH. (**B**) P70S6K phosphorylation was measured using ELISA. (**C**) SK1 activity was measured using radiolabelling. (**D**) SK1 expression was determined by qRT-PCR, normalised against housekeeping genes (GAPDH, YWHAZ and UBC) and analysed using qBase software. Columns, mean of three independent experiments performed in triplicate; bars, SEM. ($^{*}P < 0.05$; $^{**}P < 0.01$; $^{\S}P < 0.001$; NS, not significant, $P > 0.05$).

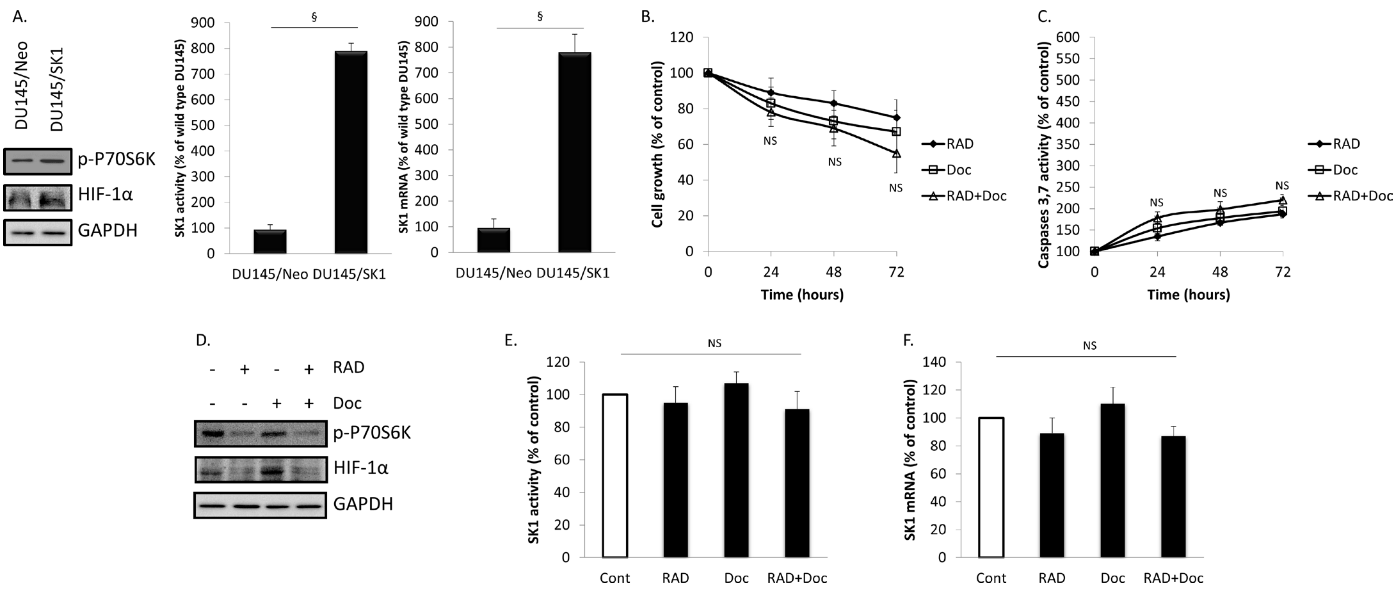

Supplementary Figure S3: Overexpression of SK1 restores DU145 prostate cancer cells chemoresistance. (**A**) Cell extracts for DU145/Neo and DU145/SK1 were loaded on acrylamide mini gel and probed for p-P70S6K, HIF-1α and GAPDH; SK1 activity and SK1 expression were measured using radiolabelling and qRT-PCR, respectively. DU145/SK1 cells were starved overnight then incubated with 0.1% DMSO (Cont), 100 nM RAD001 (RAD), 5 nM docetaxel (Doc) and the combination of these drugs (RAD + Doc) for 72 h (**B**, **C**) or 24 h (**D**–**F**). (B) Cell proliferation was measured using MTT assay. (C) Activity of caspases 3,7 was measured using caspases assay. (D) Cell extracts were loaded on acrylamide mini gel and probed for p-P70S6K, HIF-1α and GAPDH. E. SK1 activity was measured using radiolabelling. F. SK1 expression was determined by qRT-PCR, normalised against housekeeping genes (GAPDH, YWHAZ and UBC) and analysed using qBase software. Columns, mean of three independent experiments performed in triplicate; bars, SEM. ($^{*}P < 0.05$; $^{**}P < 0.01$; $^{\S}P < 0.001$; NS, not significant, $P > 0.05$).

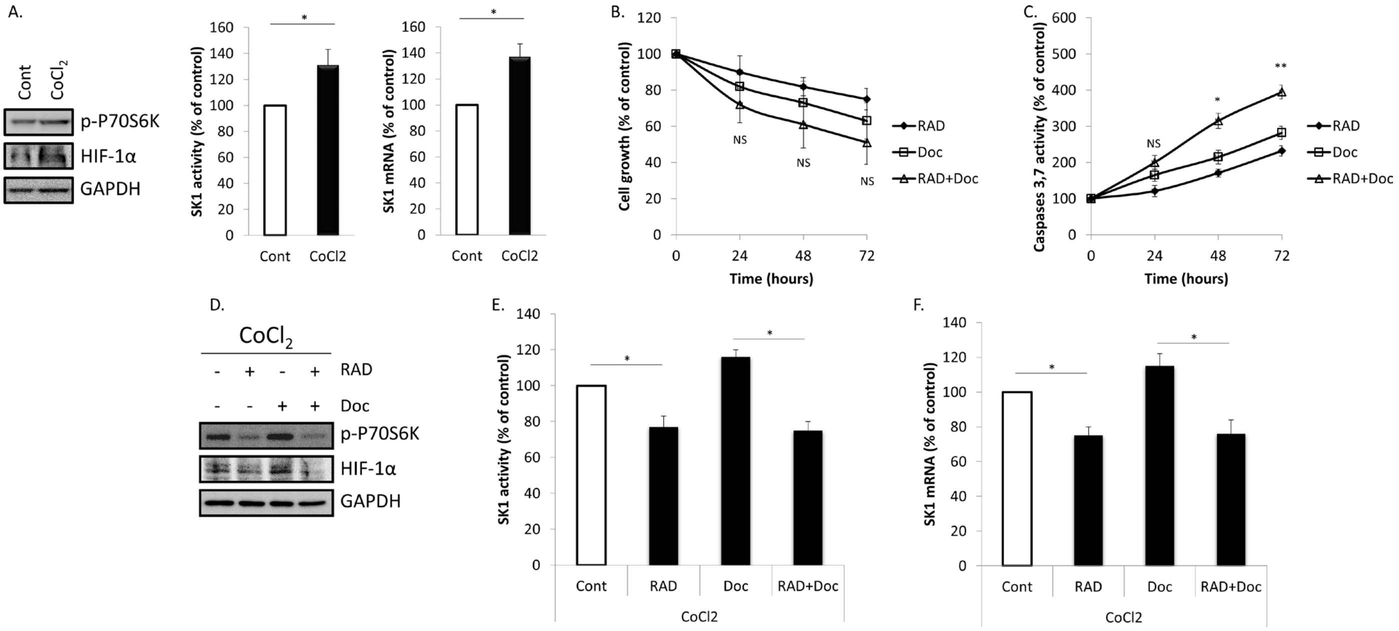

Supplementary Figure S4: Treatment with $CoCl_2$ partially restores DU145 prostate cancer cells chemoresistance and SK1 expression reduced by RAD001. (**A**) DU145 cells were treated with or without 100 μM of CoCl2 for 24 h. Cell extracts were loaded on acrylamide mini gel and probed for p-P70S6K, HIF-1α and GAPDH; SK1 activity and SK1 expression were measured using radiolabelling and qRT-PCR, respectively. DU145 cells were pretreated for 1 h with 100 μM of CoCl2 then treated with 0.1% DMSO (Cont), 100 nM RAD001 (RAD), 5 nM docetaxel (Doc) and the combination of these drugs (RAD + Doc) for 72 h (**B**, **C**) or 24 h (**D**–**F**). (B) Cell proliferation was measured using MTT assay. (C) Activity of caspases 3,7 was measured using caspases assay. (D) Cell extracts were loaded on acrylamide mini gel and probed for p-P70S6K, HIF-1α and GAPDH. E. SK1 activity was measured using radiolabelling. (F) SK1 expression was determined by qRT-PCR, normalised against housekeeping genes (GAPDH, YWHAZ and UBC) and analysed using qBase software. Columns, mean of three independent experiments performed in triplicate; bars, SEM. ($^{*}P < 0.05$; $^{**}P < 0.01$; $^{\S}P < 0.001$; NS, not significant, $P > 0.05$).
